# Clinical characteristics and risk factors associated with mortality in patients with severe community-acquired pneumonia and type 2 diabetes mellitus

**DOI:** 10.1186/s13054-021-03841-w

**Published:** 2021-12-07

**Authors:** Dong Huang, Dingxiu He, Linjing Gong, Wen Wang, Lei Yang, Zhongwei Zhang, Yujun Shi, Zongan Liang

**Affiliations:** 1 Department of Respiratory and Critical Care Medicine, West China Hospital, Sichuan University, No. 37 Guoxue Alley, Chengdu, 610041 Sichuan China; 2 Institute of Clinical Pathology, Key Laboratory of Transplant Engineering and Immunology, NHC, West China Hospital, Sichuan University, No. 37 Guoxue Alley, Chengdu, 610041 Sichuan China; 3 Department of Emergency Medicine, The People’s Hospital of Deyang, Deyang, Sichuan China; 4 Chinese Evidence-Based Medicine Center and CREAT Group, West China Hospital, Sichuan University, Chengdu, Sichuan China; 5 Department of Critical Care Medicine, West China Hospital, Sichuan University, Chengdu, Sichuan China

**Keywords:** Severe community-acquired pneumonia, Type 2 diabetes mellitus, Clinical characteristics, Risk factors, Nomogram

## Abstract

**Background:**

The present study was performed to investigate the impacts of type 2 diabetes mellitus (T2DM) on severe community-acquired pneumonia (SCAP) and to develop a novel prediction model for mortality in SCAP patients with T2DM.

**Methods:**

This was a retrospective observational study conducted in consecutive adult patients with SCAP admitted to the intensive care unit (ICU) of West China Hospital, Sichuan University, China, between September 2011 and September 2019. The primary outcome was hospital mortality. A propensity score matching (PSM) analysis model with a 1:2 ratio was used for the comparisons of clinical characteristics and outcomes between T2DM and nondiabetic patients. The independent risk factors were identified via univariate and then multivariable logistic regression analysis and were then used to establish a nomogram.

**Results:**

In total, 1262 SCAP patients with T2DM and 2524 matched patients without T2DM were included after PSM**.** Patients with T2DM had longer ICU length of stay (LOS) (13 vs. 12 days, *P* = 0.016) and higher 14-day mortality (15% vs. 10.8%, *P* < 0.001), 30-day mortality (25.7% vs. 22.7%, *P* = 0.046), ICU mortality (30.8% vs. 26.5%, *P* = 0.005), and hospital mortality (35.2% vs. 31.0%, *P* = 0.009) than those without T2DM. In SCAP patients with T2DM, the independent risk factors for hospital mortality were increased numbers of comorbidities and diabetes-related complications; elevated C-reactive protein (CRP), neutrophil to lymphocyte ratio (NLR), brain natriuretic peptide (BNP) and blood lactate; as well as decreased blood pressure on admission. The nomogram had a C index of 0.907 (95% CI: 0.888, 0.927) in the training set and 0.873 (95% CI: 0.836, 0.911) in the testing set, which was superior to the pneumonia severity index (PSI, AUC: 0.809, 95% CI: 0.785, 0.833). The calibration curve and decision curve analysis (DCA) also demonstrated its accuracy and applicability.

**Conclusions:**

SCAP patients with T2DM had worse clinical outcomes than nondiabetic patients. The nomogram has good predictive performance for hospital mortality and might be generally applied after more external validations.

**Supplementary Information:**

The online version contains supplementary material available at 10.1186/s13054-021-03841-w.

## Background

Community-acquired pneumonia (CAP) is a leading cause of infection among adults worldwide and is associated with high rates of hospitalization and hospital length of stay (LOS) [1]. The most common pathogens include *human rhinovirus, influenza virus, Streptococcus pneumoniae,* and *Staphylococcus aureus* [2]. According to the Infectious Diseases Society of America/American Thoracic Society (IDSA/ATS) consensus guidelines, severe community-acquired pneumonia (SCAP) was defined as fulfilment of at least 1 major criterion (septic shock with need for vasopressors; respiratory failure requiring mechanical ventilation) or 3 minor criteria (respiratory rate ≥ 30 breaths/min; PaO_2_/FiO_2_ ratio ≤ 250; multilobar infiltrates; confusion/disorientation; blood urea nitrogen level ≥ 20 mg/dL; white blood cell count < 4000 cells/µL; platelet count < 100,000/µL; core temperature < 36 °C; hypotension requiring aggressive fluid resuscitation) [3]. The mortality related to SCAP has barely changed during the past decades and remains a major concern despite advances in vaccine strategies and rapid diagnostic tests, appropriate and adequate antibiotic coverage, and earlier ventilatory support [4]. Cavallazzi et al. conducted a prospective population-based cohort study including 7449 patients in the USA. They reported that 23% of CAP patients required intensive care, and their mortality rates were 27% at 30 days and 47% at one year [5].

Type 2 diabetes mellitus (T2DM) also places a huge burden on health care systems, with globally increased incidence and prevalence in recent years. It is estimated that 500 million people live with T2DM worldwide [6]. Most prior studies have revealed that individuals with diabetes are at increased risk of CAP and adverse outcomes after CAP, including short-term and long-term mortality rates [7–9]. However, one retrospective study including 354 cases of pneumonia in Saudi Arabia also found that there was no significant difference in mortality between diabetic and nondiabetic CAP patients [10], which might be due partly to the heterogeneous study designs, sample sizes, and potential unadjusted confounding factors. Previous studies have also found that, compared with CAP patients without diabetes, those with diabetes were older and had more comorbidities, increased rates of development of pleural effusion, and mortality. However, there were no significant differences in etiology [11].

Considering that little is known about SCAP in T2DM patients, additional studies in different areas and patients are required to further investigate their clinical features and prognosis, which might be beneficial for treatment options. Moreover, considering the high prevalence of T2DM among people with SCAP, early evaluation, risk stratification and prediction of mortality might be crucial for improving prognosis. The CURB-65 score, including confusion, urea > 7 mmol/L, respiratory rate ≥ 30 breaths/min, systolic blood pressure < 90 mm Hg or diastolic blood pressure ≤ 60 mm Hg, and age ≥ 65 years, and pneumonia severity index (PSI), including age, nursing home residence, coexisting illnesses, physical examination findings, and laboratory and radiographic findings, are the two most widely used severity scores for pneumonia. However, their accuracy and applicability are decreased in T2DM patients [12]. Hence, there is an urgent need for novel, reliable and convenient predictive tools.

The present study was performed to explore the associations between T2DM and outcomes of SCAP, as well as the risk factors and a novel prediction model for hospital mortality in patients with SCAP and T2DM.

## Methods

### Study design and participants

This was a retrospective observational study conducted in a 172-bed medical intensive care unit (ICU) of a large tertiary care teaching hospital in Sichuan Province, China. It was performed in accordance with the amended Declaration of Helsinki. Ethics approval was obtained from the West China Hospital of Sichuan University Biomedical Research Ethics Committee (No. 2021-828). Written informed consent was waived due to the retrospective noninterventional design.

With approximately 20–30 variables potentially associated with hospital mortality in SCAP patients with T2DM, the minimum sample size required 200–300 deaths to follow the principle of at least ten outcome events per variable in the regression analysis [13]. Considering that the mortality of SCAP was approximately 30%, the sample size of patients with SCAP and T2DM was estimated to be approximately 1000. In addition, 5000 SCAP patients were needed because the incidence of T2DM in SCAP patients was approximately 20% according to previous reports [7–11]. All consecutive adult patients with a diagnosis of SCAP admitted to the ICU between September 2011 and September 2019 were enrolled. SCAP was defined according to the IDSA/ATS guidelines [3].

The exclusion criteria were as follows: (1) under 18 years old; (2) being pregnant; (3) residents of long-term care facilities/nursing homes; (4) prior hospitalization within 30 days of study enrollment; (5) discharged or having incomplete data within 24 h of admission; (6) severe immunosuppression: human immunodeficiency virus infection, autoimmune diseases, chemotherapy, or other immunosuppressive therapy; and (7) prediabetes conditions, type 1 diabetes mellitus (T1DM) or other nonspecific types of diabetes.

Only the first admission was included if the patient had multiple admissions during the study period. The diagnosis of T2DM was based on medical records of previous clinical and/or biochemical diagnosis, self-reported diagnosis confirmed by medical records reviewed by physicians, or use of antidiabetic medicine (oral antidiabetic agents or insulin). T2DM was defined according to the American Diabetes Association guidelines [14]. Diabetes-related complications included retinopathy, nephropathy, vasculopathy and peripheral neuropathy. All patients received standard care and antimicrobial agents at the discretion of the physician in charge and based on the recommendations of the CAP guidelines [3]. The occurrence of diabetic ketoacidosis (DKA) or hyperglycemic hyperosmolar status (HHS) during hospitalization was also diagnosed by the physician in charge.

### Study outcomes and data collection

The following clinical data within 24 h of admission to the ICU were collected anonymously from electronic medical records: demographics, comorbidities, primary symptoms and vital signs on admission, as well as laboratory examinations (hematological data, biochemical parameters, inflammatory markers, coagulation indicators, etc.). The most severe value was recorded for analysis if any laboratory examination was repeated more than once within 24 h of admission. The PSI at admission, which had been demonstrated to have a higher discriminative power in predicting mortality than CURB-65, was also used as a severity score [15].

Continuous variables were categorized for further analysis and the development of a prediction model. The threshold value of each continuous variable was determined by the clinically relevant cutoff value or upper limit or lower limit of the normal range. Two trained clinicians reviewed the medical records and completed the data collection independently. Any disagreement was resolved by a third doctor and team discussion until consensus was reached.

Patient follow-up was until hospital discharge. The primary outcome was hospital mortality, and the secondary outcomes included ICU LOS, hospital LOS, ICU mortality, and 14-day and 30-day mortality after the diagnosis of SCAP.

### Statistical analysis

Data were all analyzed using IBM SPSS Statistical version 23.0 (SPSS, Chicago, IL, USA) and R software 4.0.2 (R Foundation for Statistical Computing). A two-sided *p* < 0.05 was considered statistically significant. Age, sex and comorbidities are generally significantly different between CAP patients with and without diabetes and are thought to be risk factors for disease severity and death from CAP [16]. In particular, the comorbidities included cancer, hypertension, chronic hematological diseases, hepatic diseases, renal diseases, cardiovascular diseases, pulmonary diseases and cerebrovascular diseases. Therefore, a propensity score matching (PSM) analysis model with a caliper of 0.2 was used to balance differences in the above variables between groups, eliminate possible selection bias and increase the evidence level of the retrospective study. A PSM ratio of 1:2 was achieved via the “nearest‐neighbor” matching method to select statistically matched pairs of SCAP patients according to age, sex and comorbidities.

Multiple imputation (MI) was used to account for missing data if the missing values were less than 20%, and variables with a missing rate of more than 20% were excluded. MI was performed by using Bayesian methods in SPSS. The data were tested using the Kolmogorov–Smirnov normality test and Bartlett’s test for homogeneity of variance. Data are described as the counts (%) for categorical variables and either the means ± standard deviation (SD) or medians (interquartile range, IQR) for continuous variables as appropriate. The Mann–Whitney U test, Fisher’s exact test and chi-square analysis were used to test for differences between groups as appropriate. Kaplan–Meier plots with log-rank statistics were used to assess differences in survival between the propensity score-matched T2DM and non-T2DM groups. Survival analysis in SCAP patients with T2DM was also conducted to explore the impacts of diabetes-related complications and DKA or HHS on prognosis.

Then, the SCAP patients with T2DM were randomly divided into a training set (70% of patients) and a testing set (30%). The training set was applied to develop a prediction model, and the testing set was used to validate the performance of the model. In the training set, variables associated with mortality in the univariate logistic regression analysis were included in the multivariable analysis to identify independent risk factors. The results were reported as odds ratios (ORs) and 95% confidence intervals (95% CIs). The prediction model was developed through the “rms” package in R based on the results of multivariate logistic regression. Then, a nomogram was established based on the prediction model.

We used the concordance index (C index) with 95% CI, receiver operating characteristic (ROC) curve analysis, calibration curve and decision curve analysis (DCA) to assess the goodness of fit, accuracy and applicability of the predictive nomogram in the training and testing sets [17–19]. Meanwhile, ROC curve analysis of PSI was also performed to compare its predictive capacity for hospital mortality with our nomogram. These results are reported as the area under the curve (AUC) and 95% CI.

## Results

### PSM and clinical characteristics among SCAP patients

**Fig. 1:**
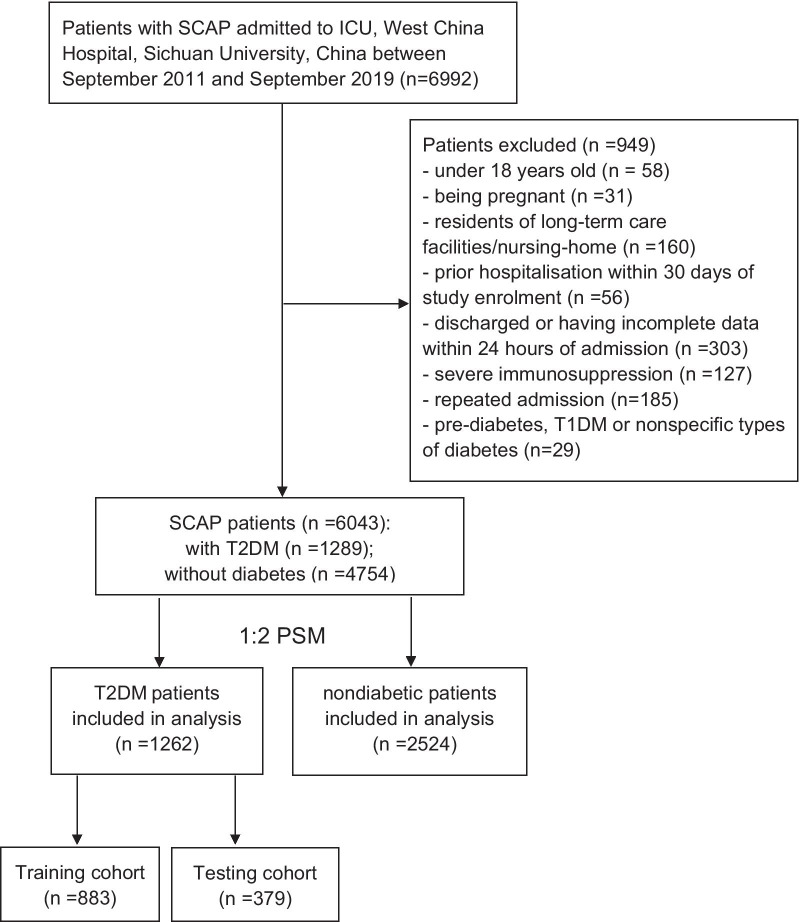
Study population. SCAP: severe community-acquired pneumonia; ICU: intensive care unit; T1DM: type 1 diabetes mellitus; T2DM: type 2 diabetes mellitus; PSM: propensity score matching

In total, 6992 patients were identified with SCAP in the present study. Then, 949 patients were excluded according to the exclusion criteria. Among the remaining 6043 SCAP patients, 1289 (21.3%) patients were found to have T2DM. Finally, 1262 patients with T2DM and 2524 matched patients without T2DM were included in our analysis after PSM (Fig. 1). A histogram matching the pre- and postpropensity scores showed that the PSM was successful (Additional file 1: Fig. S1). As shown in Table 1, covariates including age, sex and comorbidities were all balanced between the groups after matching. Their *P* values were all above 0.05, and standardized mean differences (SMDs) were all under 0.10.

**Table 1:** Comparisons of clinical characteristics and outcomes among all SCAP patients

Variables	Overall (n = 3786)	SCAP patients without T2DM (n = 2524)	SCAP patients with T2DM (n = 1262)	*P* value
Demographic characteristics				
Age (years old)	66.00 (53.00, 76.00)	66.00 (53.00, 76.00)	66.00 (53.00, 76.00)	0.934
Sex: male (%)	2438 (64.4)	1636 (64.8)	802 (63.5)	0.464
Comorbidities				
Cancer (%)	679 (17.9)	449 (17.8)	230 (18.2)	0.776
Chronic hematological diseases (%)	71 (1.9)	45 (1.8)	26 (2.1)	0.641
Chronic hepatic diseases (%)	99 (2.6)	62 (2.5)	37 (2.9)	0.45
Chronic renal diseases (%)	251 (6.6)	161 (6.4)	90 (7.1)	0.42
Chronic cardiovascular diseases (%)	606 (16)	394 (15.6)	212 (16.8)	0.37
Hypertension (%)	1050 (27.7)	704 (27.9)	346 (27.4)	0.788
Chronic pulmonary diseases (%)	919 (24.3)	597 (23.7)	322 (25.5)	0.223
Chronic cerebrovascular diseases (%)	100 (2.6)	64 (2.5)	36 (2.9)	0.641
Primary symptoms				
Fever (%)	856 (22.6)	580 (23.0)	276 (21.9)	0.467
Cough (%)	1233 (32.6)	847 (33.6)	386 (30.6)	0.071
Expectoration (%)	427 (11.3)	300 (11.9)	127 (10.1)	0.106
Dyspnea (%)	731 (19.3)	510 (20.2)	221 (17.5)	0.053
Coma (%)	751 (19.8)	528 (20.9)	223 (17.7)	0.02
Insanity (%)	73 (1.9)	54 (2.1)	19 (1.5)	0.226
Chest pain (%)	94 (2.5)	65 (2.6)	29 (2.3)	0.685
Vital signs on admission				
Respiratory rate (breath/min)	18.00 (15.00, 23.00)	18.00 (14.00, 22.00)	19.00 (16.00, 23.00)	< 0.001
Systolic blood pressure (mmHg)	130.00 (111, 148)	128.00 (113.25, 146)	130.00 (110, 148)	0.238
Diastolic blood pressure (mmHg)	72.00 (61.00, 85.00)	72.00 (61.00, 85.00)	73.00 (62.25, 83.00)	0.843
Temperature (°C)	36.60 (36.30, 37.20)	36.60 (36.30, 37.10)	36.80 (36.30, 37.40)	< 0.001
Heart rate (beat/min)	96.00 (81.00, 110)	96.00 (80.00, 108.00)	98.00 (87.00, 115.00)	< 0.001
Laboratory examinations				
Glucose (mmol/L)	7.36 (5.95, 10.86)	6.84 (5.50, 7.93)	12.61 (10.29, 15.30)	< 0.001
HbA1c (%)	5.99 (4.76, 8.69)	5.40 (4.40, 6.34)	10.08 (8.23, 12.24)	< 0.001
CRP (mg/L)	64.80 (18.5, 122.00)	61.5 (17.40, 118)	70.2 (21.9, 127)	0.002
Procalcitonin (µg/L)	0.33 (0.14, 1.08)	0.33 (0.13, 0.89)	0.35 (0.16, 1.60)	< 0.001
D-dimer (mg/L)	4.12 (2.17, 8.79)	4.12 (2.15, 8.33)	4.25 (2.20, 9.63)	0.012
Lactate (mmol/L)	1.40 (1.10, 2.20)	1.40 (1, 2.1)	1.60 (1.10, 2.40)	< 0.001
BNP (pg/mL)	1099 (329,3902)	978 (290, 3570)	1339 (397, 5067)	< 0.001
APTT (s)	31.80 (27.60, 38.40)	31.60 (27.40, 37.80)	32.40 (28.02, 39.48)	0.004
PT (s)	12.90 (11.90, 14.50)	12.80 (11.80, 14.30)	13.20 (12.10, 14.90)	< 0.001
Fibrinogen (g/L)	3.57 (2.64, 4.77)	3.52 (2.60, 4.64)	3.82 (2.77, 4.96)	< 0.001
INR	1.12 (1.03, 1.27)	1.11 (1.02, 1.25)	1.15 (1.05, 1.29)	< 0.001
White blood cell (×10^9^/L)	9.77 (6.72, 13.81)	9.30 (6.55, 13.31)	10.77 (7.14, 14.69)	< 0.001
Hemoglobin (g/L)	109.00 (89.00, 129)	111.00 (90.00, 131)	106.00 (86.00, 125)	< 0.001
Neutrophil (×10^9^/L)	7.81 (5.01, 11.54)	7.70 (5.01, 11.31)	8.06 (5.03, 12.18)	0.009
Lymphocyte (×10^9^/L)	0.90 (0.56, 1.35)	0.90 (0.57, 1.34)	0.90 (0.55, 1.35)	0.995
Platelet (×10^9^/L)	175.00 (111, 254)	178 (114, 256)	165 (103, 252)	0.021
Monocyte (×10^9^/L)	0.42 (0.27, 0.63)	0.43 (0.28, 0.63)	0.40 (0.23, 0.62)	0.001
ALT (IU/L)	22.00 (14.00, 43.00)	21.50 (13.00, 41.00)	23.00 (14.00, 45.00)	0.012
AST (IU/L)	29.00 (20.00, 51.00)	28.00 (20.00, 49.00)	30.00 (20.00, 55.00)	0.045
Creatinine (µmol/L)	73.00 (55, 107.25)	71.00 (54.00, 98.00)	79.00 (58.00, 133.75)	< 0.001
Uric acid (µmol/L)	236.00 (148, 343)	232.80 (144, 336)	241.00 (156, 364)	0.002
Albumin (g/L)	32.10 (28.00, 37.58)	32.7 (28.40, 38.50)	30.8 (27.30, 35.77)	< 0.001
Globulin (g/L)	25.20 (21.60, 29.00)	25.30 (21.70, 29.02)	25.00 (21.50, 28.90)	0.255
BUN (mmol/L)	7.10 (5.00, 11.40)	6.68 (4.71, 10.17)	8.55 (5.56, 14.65)	< 0.001
Total bilirubin (µmol/L)	11.80 (7.90, 17.80)	12.2 (8.2, 18.2)	10.8 (7.12, 16.7)	< 0.001
Direct bilirubin (µmol/L)	5.50 (3.50, 9.00)	5.40 (3.50, 9.00)	5.60 (3.50, 8.90)	0.647
Clinical outcomes				
ICU LOS (days)	12.00 (6.00, 23.00)	12.00 (5.00, 22.00)	13.00 (6.00, 24.00)	0.016
Hospital LOS (days)	21.00 (12.00, 33.00)	21.00 (13.00, 32.00)	20.50 (11.00, 34.00)	0.168
Need for CPR during hospitalization (%)	120 (3.2)	72 (2.9)	48 (3.8)	0.115
Death within 14 days (%)	461 (12.2)	272 (10.8)	189 (15)	< 0.001
Death within 30 days (%)	898 (23.7)	574 (22.7)	324 (25.7)	0.046
ICU mortality (%)	1058 (27.9)	669 (26.5)	389 (30.8)	0.005
Hospital mortality (%)	1226 (32.4)	782 (31.0)	444 (35.2)	0.009

Data are shown as median with interquartile range (IQR) for continuous variables or number with percentage for categorical variables

*SCAP* Severe community-acquired pneumonia, *n* numbers, *T2DM* type 2 diabetes mellitus, *CRP* C-reactive protein, *BNP* brain natriuretic peptide, *APTT* activated partial thromboplastin time, *PT* prothrombin time, *INR* international normalized ratio, *ALT* alanine aminotransferase, *AST* aspartate aminotransferase, *BUN* blood urea nitrogen, *ICU* intensive care unit, *LOS* length of stay, *CPR* cardiac pulmonary resuscitation

When comparing clinical characteristics at admission, patients with T2DM were more likely to have a higher respiratory rate (19 vs. 18 breath/min, *P* < 0.001), heart rate (98 vs. 96 beat/min, *P* < 0.001), C-reactive protein (CRP, 70.2 vs. 61.5 mg/L, *P* = 0.002), procalcitonin (PCT, 0.35 vs. 0.33 µg/L, *P* < 0.001), D-dimer (4.25 vs. 4.12 mg/L, *P* = 0.012), brain natriuretic peptide (BNP, 1339 vs. 978 pg/mL, *P* < 0.001), and blood lactate (1.6 vs. 1.4 mmol/L, *P* < 0.001) but lower hemoglobin (106 vs. 111 g/L, *P* < 0.001), platelet (165 vs. 178 × 10^9^/L, *P* = 0.021), albumin (30.8 vs. 32.7 g/L, *P* < 0.001), and total bilirubin (10.8 vs. 12.2 µmol/L, *P* < 0.001). Moreover, substantial differences in clinical outcomes were observed between the two groups. Compared with patients without T2DM, patients with T2DM had a longer ICU LOS (13 vs. 12 days, *P* = 0.016) and a higher 14-day mortality (15% vs. 10.8%, *P* < 0.001), 30-day mortality (25.7% vs. 22.7%, *P* = 0.046), ICU mortality (30.8% vs. 26.5%, *P* = 0.005), and hospital mortality (35.2% vs. 31.0%, *P* = 0.009)**.** However, the hospital LOS and need for CPR (cardiac pulmonary resuscitation) during hospitalization were not significantly different between the two groups.

**Fig. 2:**
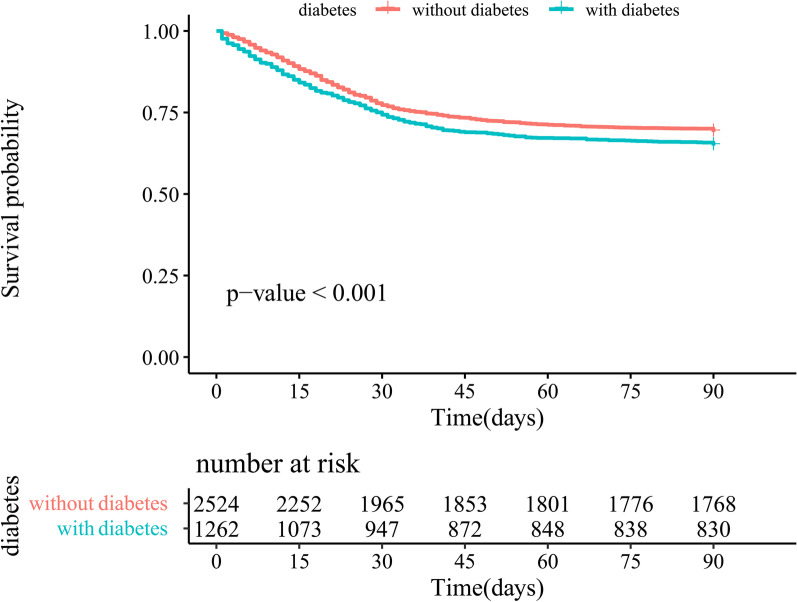
Survival curves of SCAP patients after matching

**Fig. 3:**
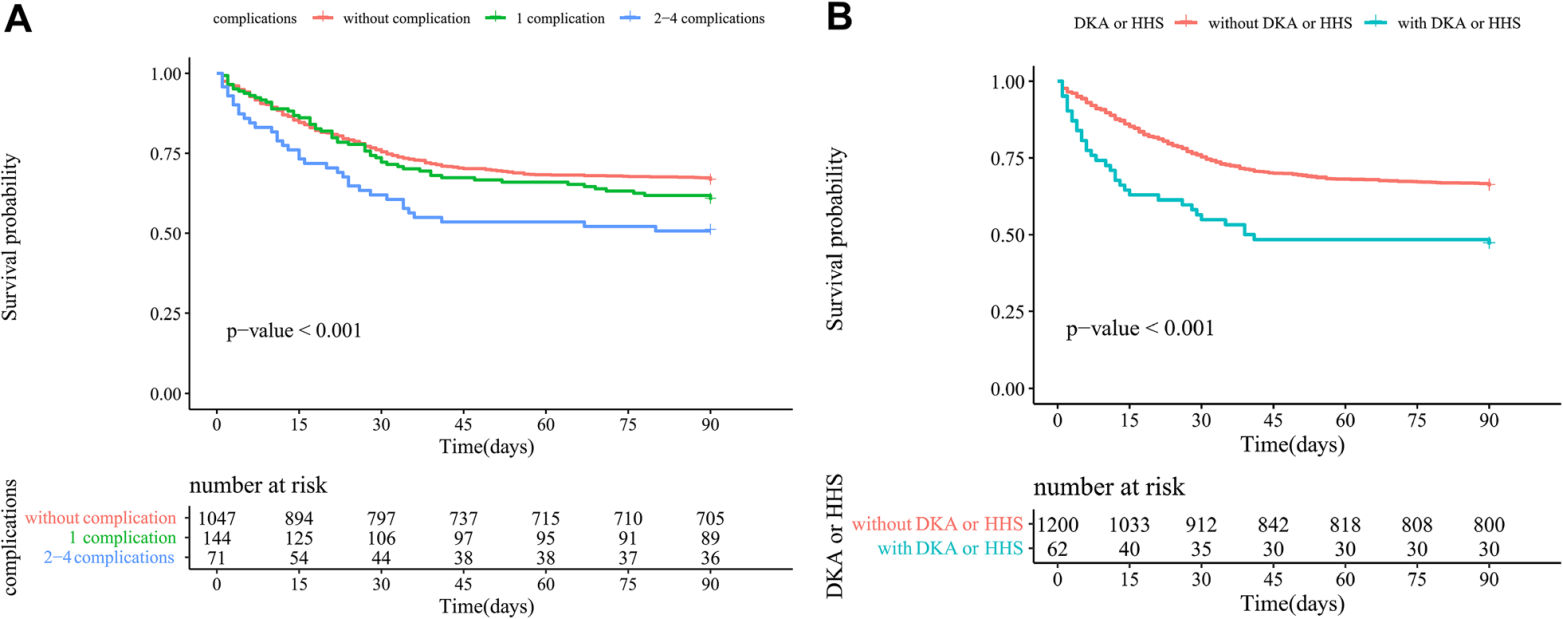
Survival curves of SCAP patients with T2DM. A: impacts of pre-admission diabetes-related complications on survivals; B: impacts of DKA or HHS during hospitalization on survivals; DKA: diabetic ketoacidosis; HHS: hyperglycemic hyperosmolar status

Among all SCAP patients, T2DM was significantly associated with poorer survival (*P* < 0.001) in the Kaplan–Meier curves (Fig. 2). Furthermore, long-term diabetes-related complications prior to hospital admission and the occurrence of DKA or HHS during hospitalization both had considerable adverse impacts on the prognosis of T2DM patients with SCAP (*P* < 0.001) (Fig. 3).

### Development of a prediction model in SCAP patients with T2DM

**Fig. 4:**
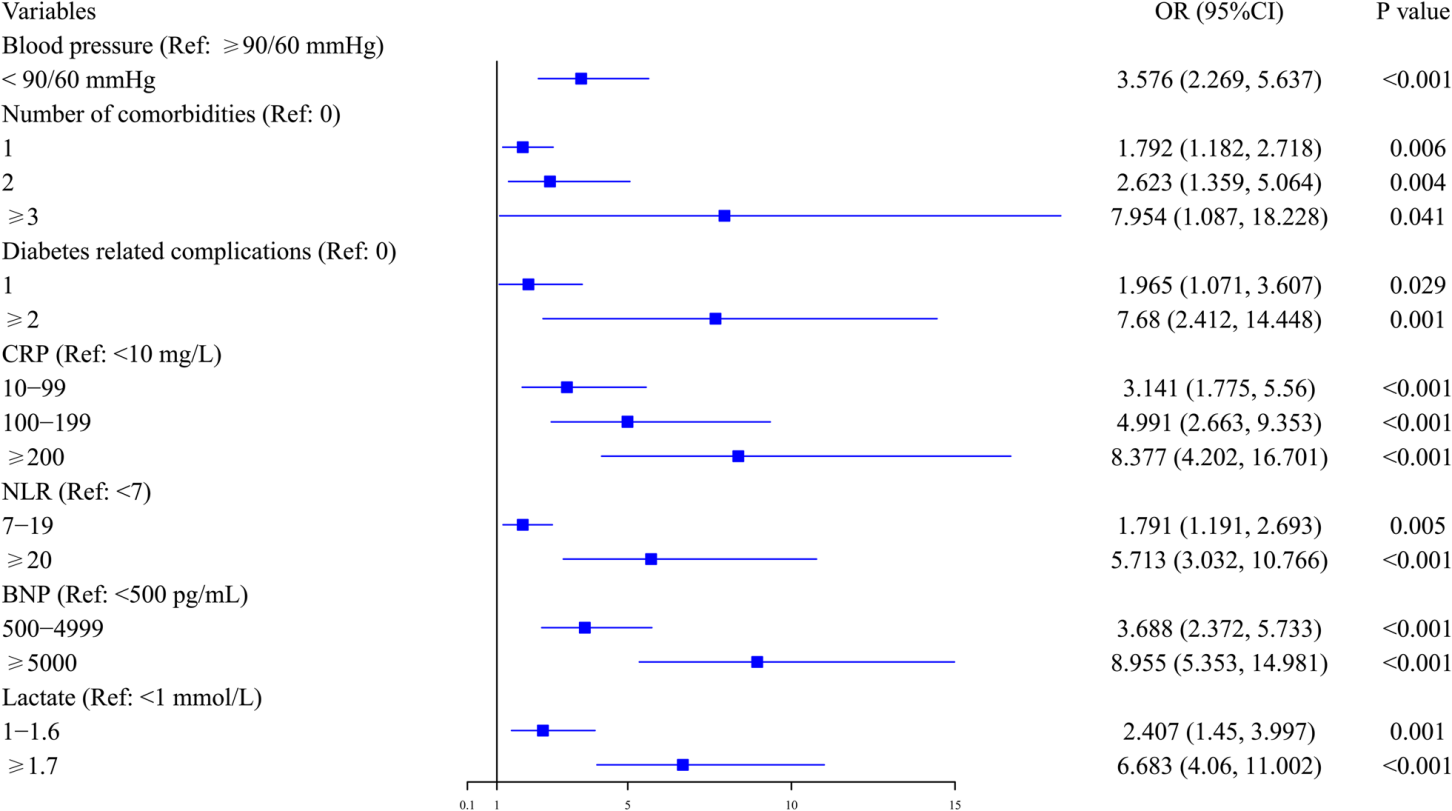
Independent risk factors for hospital mortality in the training set. Ref: reference; OR: odds ratio; 95% CI: 95% confidence interval. CRP: C-reactive protein; NLR: neutrophil to lymphocyte ratio; BNP: brain natriuretic peptide

Among the 1262 T2DM patients, 883 patients were randomized to the training set, and 379 patients were included in the testing set. In the training set, 315 (35.7%) patients eventually died. In the univariate logistic regression analysis, 17 variables were found to be associated with hospital mortality. The ORs with 95% CI are shown in Table 2. However, in the multivariate analysis, only 7 factors were independent risk factors for hospital mortality: increased numbers of comorbidities and diabetes-related complications; elevated CRP, neutrophil to lymphocyte ratio (NLR, calculated by division of neutrophils by lymphocytes measured in peripheral blood), BNP and blood lactate; as well as decreased blood pressure. The ORs with 95% CI are shown in Fig. 4.

**Table 2:** The risk factors for hospital mortality in univariate logistic regression analysis among SCAP patients with T2DM in training set

Variables	Survival (n = 568)	Death (n = 315)	OR (95%CI)	*P* value
Age	65 (53.74)	71 (59.79)	1.202 (1.089, 1.328) (per increased 10 years old)	< 0.001
Number of comorbidities				
0	369 (65)	110 (34.9)	Ref	
1	159 (28)	134 (42.5)	2.827 (2.067, 3.867)	< 0.001
2	38 (6.7)	61 (19.4)	5.385 (3.408, 8.510)	< 0.001
≥ 3	2 (0.4)	10 (3.2)	16.773 (3.621, 77.695)	< 0.001
Diabetes-related complications				
0	504 (88.7)	247 (78.4)	Ref	
1	56 (9.9)	43 (13.7)	1.567 (1.024, 2.398)	0.039
≥ 2	8 (1.4)	25 (7.9)	6.377 (2.835, 14.342)	< 0.001
Cough				
No	435 (76.6)	190 (60.3)	Ref	
Yes	133 (23.4)	125 (39.7)	2.152 (1.598, 2.898)	< 0.001
Dyspnea				
No	499 (87.9)	238 (75.6)	Ref	
Yes	69 (12.1)	77 (24.4)	2.340 (1.633, 3.353)	< 0.001
Blood pressure (mmHg)				
≥ 90/60	479 (84.3)	201 (63.8)	Ref	
< 90/60	89 (15.7)	114 (36.2)	3.052 (2.211, 4.214)	< 0.001
Heart rate (beat/min)				
< 100	332 (58.5)	145 (46)	Ref	
≥ 100	236 (41.5)	170 (54)	1.649 (1.250, 2.177)	< 0.001
CRP (mg/L)				
< 10	199 (35)	24 (7.6)	Ref	
10–99	234 (41.2)	106 (33.7)	3.756 (2.320, 6.080)	< 0.001
100–199	97 (17.1)	78 (24.8)	6.668 (3.972, 11.191)	< 0.001
≥ 200	38 (6.7)	107 (34)	23.348 (13.304, 40.975)	< 0.001
NLR				
< 7	317 (55.8)	86 (27.3)	Ref	
7–19	226 (39.8)	142 (45.1)	2.316 (1.686, 3.182)	< 0.001
≥ 20	25 (4.4)	87 (27.6)	12.827 (7.745,21.246)	< 0.001
BNP (pg/mL)				
< 500	365 (64.3)	58 (18.4)	Ref	
500–4999	149 (26.2)	123 (39)	5.195 (3.604, 7.489)	< 0.001
≥ 5000	54 (9.5)	134 (42.5)	15.616 (10.259, 23.772)	< 0.001
Lactate (mmol/L)				
< 1	213 (37.5)	50 (15.9)	Ref	
1–1.6	244 (43)	85 (27)	1.484 (1.00, 2.202)	0.05
≥ 1.7	111 (19.5)	180 (57.1)	6.908 (4.685, 10.186)	< 0.001
Platelet (×10^9^/L)				
≥ 100	463 (81.5)	215 (68.3)	Ref	
< 100	105 (18.5)	100 (31.7)	2.051 (1.492, 2.819)	< 0.001
Procalcitonin (µg/L)				
< 0.5	349 (61.4)	146 (46.3)	Ref	
0.5–1.9	112 (19.7)	77 (24.4)	1.643 (1.160,2.329)	0.005
≥ 2	107 (18.8)	92 (29.2)	2.055 (1.464,2.885)	< 0.001
APTT (s)				
< 37	402 (70.8)	199 (63.2)	Ref	
≥ 37	166 (29.2)	116 (36.8)	1.412(1.054,1.890)	0.021
AST (IU/L)				
< 35	333 (58.6)	161 (51.1)	Ref	
≥ 35	235 (41.4)	154 (48.9)	1.355 (1.028, 1.788)	0.031
Creatinine (µmol/L)				
< 100	362(63.7)	173(54.9)	Ref	
≥ 100	206(36.3)	142(45.1)	1.442(1.090,1.909)	0.010
BUN (mmol/L)				
< 7	253 (44.5)	90 (28.6)	Ref	
≥ 7	315 (55.5)	225 (71.4)	2.008 (1.495, 2.697)	< 0.001

Data are shown as median with number with percentage for categorical variables

*SCAP* Severe community-acquired pneumonia, *n* numbers, *OR* odds ratio, *95% CI* 95% confidence interval, *CRP* C-reactive protein, *NLR* neutrophil to lymphocyte ratio, *BNP* brain natriuretic peptide, *APTT* activated partial thromboplastin time, *AST* aspartate aminotransferase, *BUN* blood urea nitrogen, *Ref* reference

**Fig. 5:**
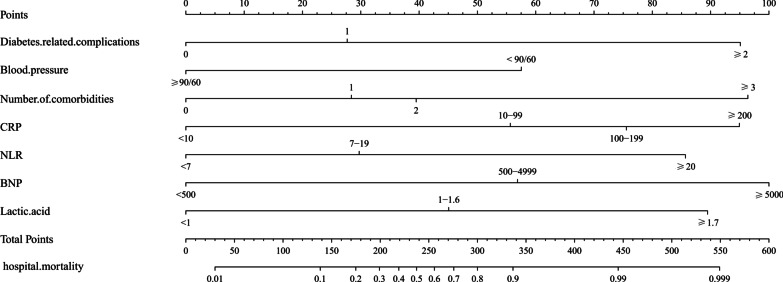
The nomogram for hospital mortality in SCAP patients with T2DM. Blood pressure (mmHg); CRP: C-reactive protein (mg/L); NLR: neutrophil to lymphocyte ratio; BNP: brain natriuretic peptide (pg/mL); blood lactate (mmol/L)

As a result, these seven factors were included in the prediction model, as described in Fig. 5. Each predictive factor was assigned a single score, which is presented on the top line of the nomogram. The total score of each patient is the sum of each single score. On the bottom of the nomogram, the probabilities of hospital mortality in SCAP patients with T2DM were predicted in terms of the total scores.

### Evaluation and validation of prediction model

**Fig. 6:**
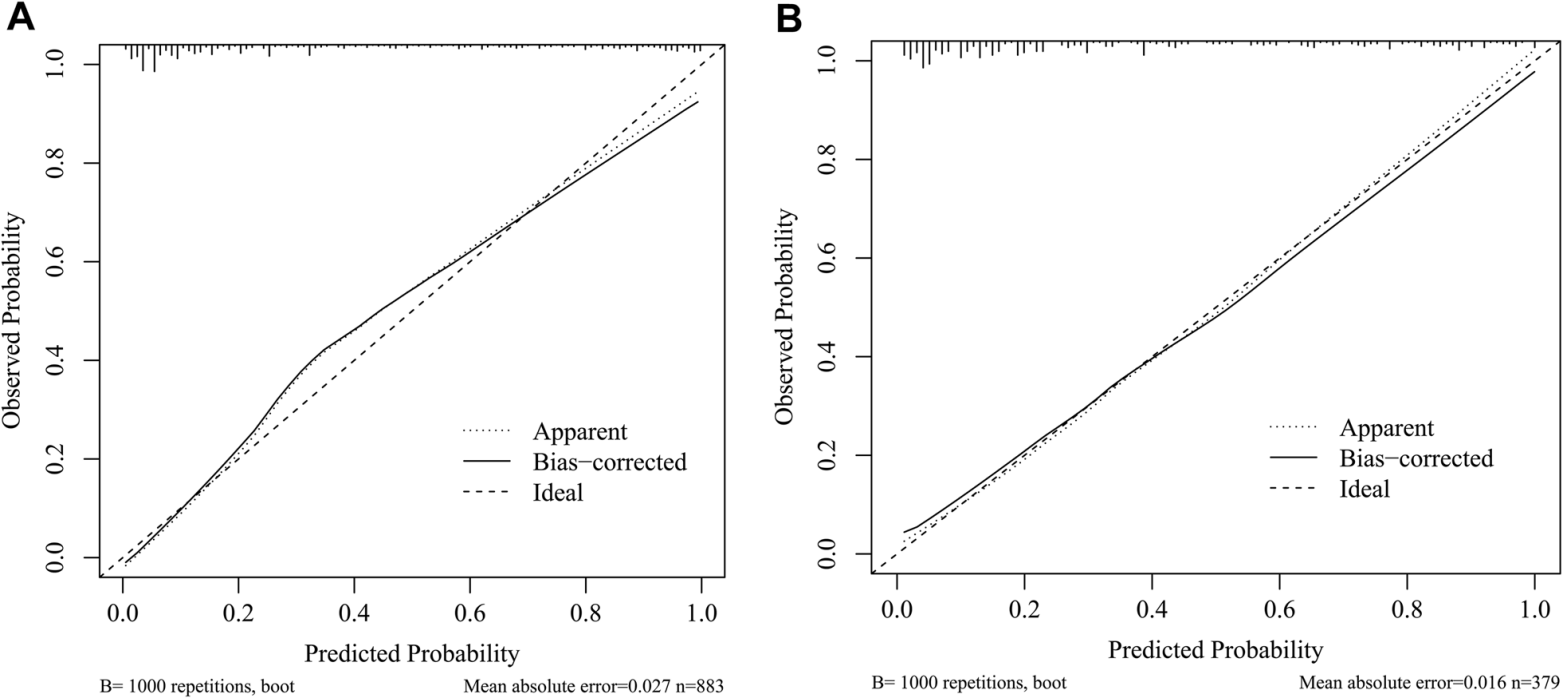
Calibration curves of nomogram. A: training set; B: testing set

**Fig. 7:**
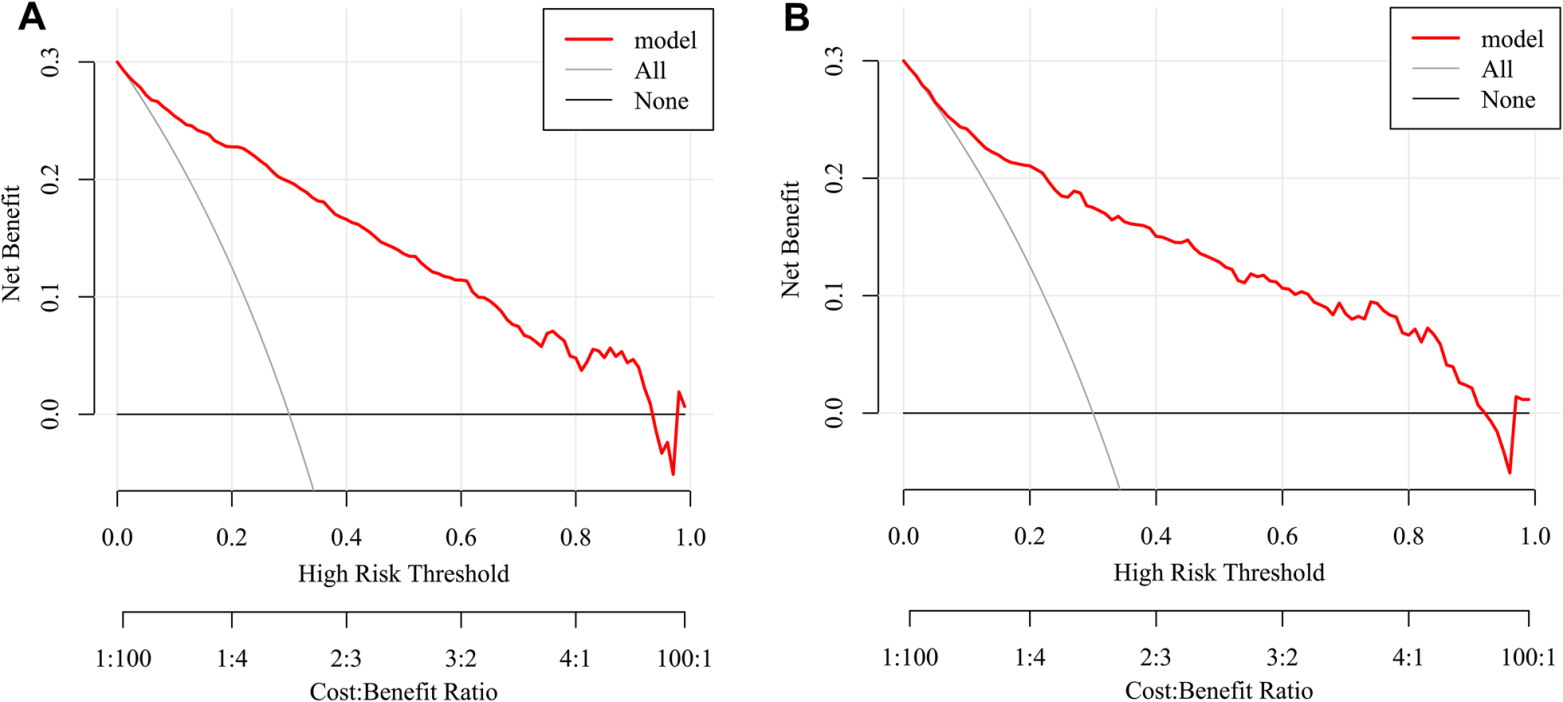
DCA of nomogram. A: training set; B: testing set. DCA: decision curve analysis

Using the bootstrap method, the C index was 0.907 (95% CI: 0.888, 0.927) in the training set, which indicated that our nomogram had good predictive value. The ROC curve is shown in Additional file 1: Fig. S2A. In Fig. 6A, the calibration curve did not significantly deviate from the reference line. There was good consistency between the predicted values by the nomogram and the actual observed values. The bias-corrected C index was 0.898. Subsequently, DCA was performed to evaluate the clinical applicability of the prediction model. As shown in Fig. 7A, DCA demonstrated that the nomogram had good overall net benefits within a wide range of threshold probabilities.

In the testing set, 129 patients (34.0%) died, and the C index was 0.873 (95% CI: 0.836, 0.911). The ROC curve is shown in Additional file 1: Fig. S2B, and the calibration curve and DCA are shown in Figs. 6B and 7B. The bias-corrected C index was 0.853. Additionally, the AUC of PSI among all SCAP patients with T2DM was 0.809 (95% CI: 0.785, 0.833), which was slightly lower than that of the nomogram (Additional file 1: Fig. S3).

## Discussion

In our study population, there was a high burden of T2DM among SCAP patients. After minimizing the effects of common confounders, of particular concern is that the existence of T2DM, the increased diabetes-related complications, and the occurrence of DKA or HHS all had significant adverse impacts on the prognosis of SCAP patients. These seven easily obtained independent risk factors are important for the early recognition and risk evaluation of patients. Then, we established and validated a predictive nomogram for hospital mortality, which was verified to be superior to PSI in T2DM patients.

The results of the current study are partly concordant with past evidence indicating the association between diabetes and outcomes of CAP. Kornum et al. demonstrated that diabetic patients had greater adjusted 30-day (RR: 1.16, 95% CI: 1.07–1.27) and 90-day mortality (RR: 1.10, 95% CI: 1.02–1.18) following pneumonia than other patients in northern Denmark [8]. Martins et al. reported that CAP episodes in patients with DM had, on average, a 0.8-day longer hospital stay (*P* < 0.0001) and significantly higher hospital mortality (15.2% vs. 13.5%, *P* = 0.002) than patients without DM in Portugal [20]. However, López-de-Andrés found that T2DM was only related to higher CAP incidence rates but was not a risk factor for death during CAP (OR 0.92, 95% CI 0.91–0.94) in Spain [21]. One possible explanation might be that the methodology applied by researchers in previous studies was not uniform. More importantly, researchers speculated that patients with diabetes were more likely to be hospitalized with less severe CAP. In our study cohort comprising only patients with SCAP, T2DM was still found to be associated with longer ICU LOS and higher ICU and hospital mortality after matching. Hence, our results might add important evidence to previous information. We further found that SCAP patients with T2DM were less likely to exhibit some classical symptoms, such as fever, cough, dyspnea, and chest pain, than those without diabetes, which is consistent with prior reports [22]. Clinicians should take into account those atypical symptoms for the early diagnosis of SCAP.

The relationship between T2DM and the prognosis of SCAP might be mediated by several factors. First, evidence has been found that the reduction in diffusion capacity in pulmonary function abnormalities and microangiopathy due to impaired pulmonary microvasculature alveolar epithelial basal lamina in diabetes might be related to more severe CAP [23]. Then, it is plausible that impaired immunocompetence, disturbances in pulmonary host defense and dysregulated pulmonary inflammatory function play a major role in the poor prognosis of SCAP. For instance, excessive or chronic activation of the NLRP3 inflammasome, an important innate immune sensor and inflammation regulator, and subsequent interleukin release are implicated in the pathogenesis of diabetes and pneumococcal pneumonia [24]. However, these conclusions are not supported by strong scientific evidence. It has also been reported that the mechanism of association between pre-existing diabetes and a higher risk of death following CAP might not be due to an altered immune response but to worsening of pre-existing cardiovascular and kidney disease [25]. In addition, some other risk factors that negatively affect immune function and host defense mechanisms, such as obesity and other lifestyle factors, might be involved [26]. Although we have demonstrated a clear association between T2DM and mortality among SCAP patients after adjusting for confounders, more research about the pathophysiological mechanisms in SCAP patients with T2DM is warranted. In addition, the true association of T2DM and SCAP needs more verification after excluding other potential confounders, such as prior use of pneumococcal vaccine, treatments of T2DM, duration of symptoms prior to admission, and time to first dose of appropriate antibiotic therapy [27].

Cheng et al. established a risk score, including NLR ≥ 4, pulse ≥ 125/min, confusion, and glucose on admission ≥ 9 mmol/L, to predict in-hospital mortality among 1360 patients with T2DM and concomitant CAP (AUC: 0.858) [28]. In another recent study, Ma et al. included 531 patients and developed a similar nomogram consisting of age, pulse, urea and albumin for predicting in-hospital mortality of CAP in patients with T2DM (AUC: 0.814, 95% CI: 0.770–0.853) [29]. However, the present study still has some strengths. First, our study had a large sample size and focused solely on ICU settings and SCAP patients, which had significantly increased systemic complications and mortality during hospitalization. Then, we included more comprehensive independent risk factors, including comorbidities, indicators of shock and cardiac insufficiency, and inflammatory and acidosis indices. It should be noted that the increased serum glucose and HbA1c levels at admission might inevitably be affected by information that was difficult to capture or remained unmeasured. In addition, they were not significantly associated with the risk of death in CAP patients with diabetes, which was shown in previous studies and the current study [30, 31]. Therefore, we speculate that prehospital T2DM-related complications might be better suited than serum glucose or HbA1c levels to reflect the chronicity and severity of diabetes and could be used to predict mortality. Additionally, compared with the two above predictive tools, the current nomogram had a higher C index and greater net benefits in DCA in both the training cohort and testing cohort.

In SCAP patients with T2DM, the predictive values of comorbidities and decreased blood pressure or signs of shock at admission are in agreement with previous reports from general CAP patients. The increased NLR during SCAP represents the ratio of increased neutrophils reflecting aggravated inflammation and systemic inflammatory storms and decreased lymphocytes representing dysregulated and compromised immune responses. It could effectively overcome the drawbacks of absolute values that may be affected by factors such as dehydration and was independently associated with hospital and ICU mortality in patients with SCAP [32]. As another common inflammatory index, the association of increased CRP at admission or sequential evaluation after admission and poor outcome of SCAP has been widely identified and confirmed. [33, 34] Christ-Crain et al. concluded that BNP, a common marker of cardiac stress and heart failure, is still a powerful and independent predictor of death and treatment failure (AUC: 0.75) in CAP after the exclusion of patients with a history of heart failure and coronary or hypertensive heart disease. Furthermore, they found that, when used in conjunction with PSI, BNP significantly improved the risk prediction compared with PSI alone (AUC 0.78 vs. 0.71; *P* = 0.02) [35]. In this situation, we might attribute increased BNP to hypoxia, leading to pulmonary vasoconstriction, pulmonary hypertension and right heart overload [36]. Meanwhile, proinflammatory cytokines could also induce BNP secretion [37]. As expected, in line with prior studies about CAP, our investigation showed that blood lactate level remained a significant prognostic factor of mortality in multivariate analysis [38, 39]. We speculate that hypoxia, circulatory disorder and hypoperfusion, and organ failure might cause increased lactate.

Based on these results, more intensive and individualized surveillance should be considered, and efforts should be made to improve the management strategies for patients admitted with T2DM in the ICU due to its significant adverse impacts on the prognosis of SCAP. For SCAP patients with T2DM, a cost-effective, convenient and accurate tool may help identify patients at increased risk of death and curb poor outcomes. However, several questions remain unanswered. There is still a knowledge gap in the available literature concerning the features of chest computed tomography (CT) images of T2DM patients, the biological mechanisms underlying the associations between DKA or HHS and outcomes of SCAP, and the clinical characteristics of SCAP patients with T1DM. Additionally, definite recommendations on the optimal cutoff values of these risk factors used in prediction models are lacking. Therefore, our nomogram needs to be updated or recalibrated in further studies.

Our study is subject to several limitations. First, this was a retrospective, single-center study with routinely collected data in a limited number of patients. A small percentage of the potential study population had no available clinical data and therefore was not included. Meanwhile, some participants might have had undetected or undiagnosed T2DM on admission. These conditions could have led to bias or misclassification in our results. Although we divided patients into a training cohort and a testing cohort, the nomogram was derived from particular periods and places where data were collected. We did not have more external validation study data for our prediction model and therefore were unable to formally determine the robustness of the results. Second, the results might have been affected by some unadjusted confounders or other risk factors, such as interventions and antibiotic therapies. Third, we lacked dynamic clinical and laboratory data and failed to perform follow-up after discharge due to the scarcity of relevant information.

## Conclusions

In summary, SCAP patients with T2DM have distinct clinical characteristics and higher mortality than nondiabetic patients. The subset of patients with increased numbers of diabetes-related complications and the development of DKA or HHS had a poor prognosis. Independent factors on admission for mortality in SCAP patients with T2DM were increased numbers of comorbidities and diabetes-related complications; elevated CRP, NLR, BNP and blood lactate; as well as decreased blood pressure. Our nomogram has good predictive performance for hospital mortality and might be generally applied after more external validations.

## Supplementary Information

**Additional file 1.** **Fig. S1** Histograms of propensity scores before and after matching. **Fig. S2** The ROC curves of nomogram. A. training set; B. testing set. ROC: receiver operating characteristic. **Fig. S3** ROC curve of PSI in SCAP patients with T2DM. ROC: receiver operating characteristic; PSI: Pneumonia Severity Index.

## Data Availability

The datasets used and/or analyzed during the current study are available from the corresponding author on reasonable request.

## Abbreviations

T2DM: Type 2 diabetes mellitus
SCAP: Severe community-acquired pneumonia
ICU: Intensive care unit
PSM: Propensity score matching
LOS: Length of stay
CRP: C-reactive protein
NLR: Neutrophil to lymphocyte ratio
BNP: Brain natriuretic peptide
PSI: Pneumonia Severity Index
AUC: Area under the curve
DCA: Decision curve analysis
T1DM: Type 1 diabetes mellitus
DKA: Diabetic ketoacidosis
HHS: Hyperglycemic hyperosmolar status
OR: Odds ratio
95% CI: 95% Confidence interval
C index: Concordance index
ROC: Receiver operating characteristic
