# Supplementary material for: Clinical characteristics and risk factors associated with mortality in patients with severe community-acquired pneumonia and type 2 diabetes mellitus
Source: Crit Care. 2021 Dec 7;25:419. doi: 10.1186/s13054-021-03841-w (PMC8650350; doi:10.1186/s13054-021-03841-w)
Supplement: Supplementary file 1 — Additional file 1. Fig. S1 Histograms of propensity scores before and after matching. Fig. S2 The ROC curves of nomogram. A. training set; B. testing set. ROC: receiver operating characteristic. Fig. S3 ROC curve of PSI in SCAP patients with T2DM. ROC: receiver operating characteristic; PSI: Pneumonia Severity Index. [file 13054_2021_3841_MOESM1_ESM.docx]

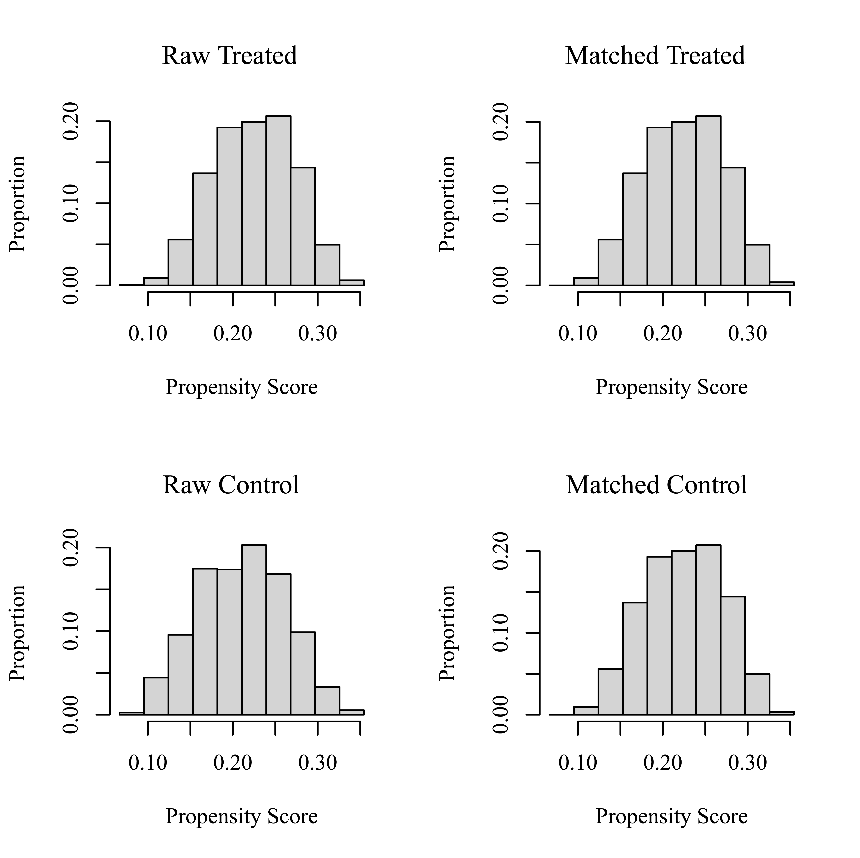


Figure S1. Histograms of propensity scores before and after matching.


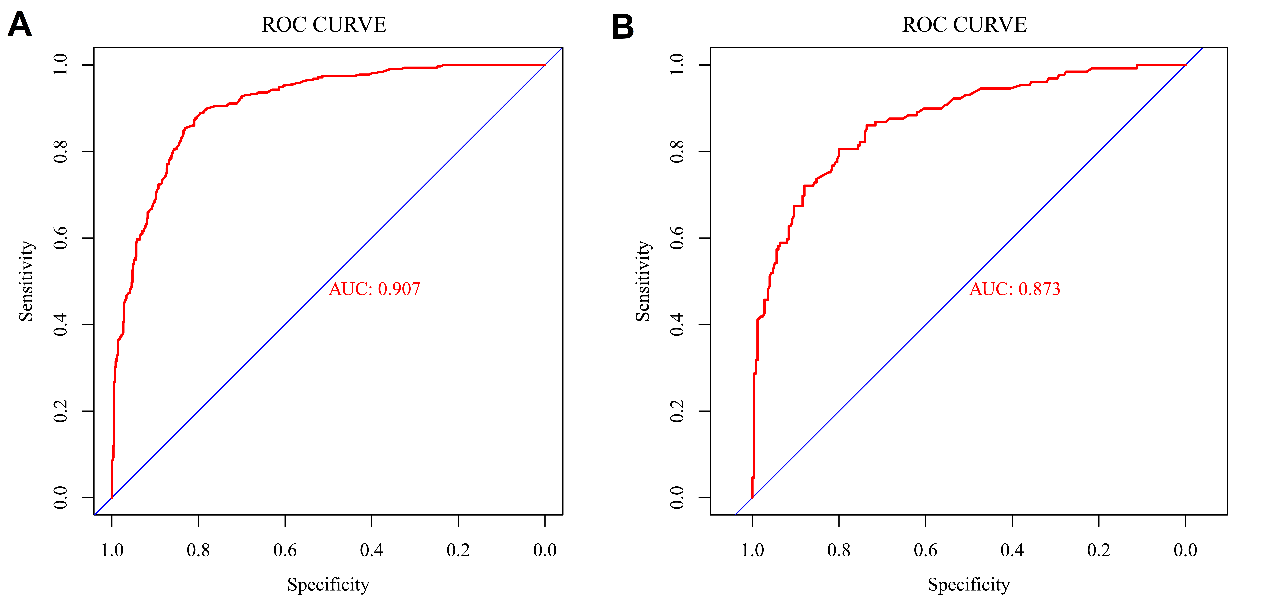


Figure S2. The ROC curves of nomogram. A. training set; B. testing set. ROC: receiver operating characteristic.


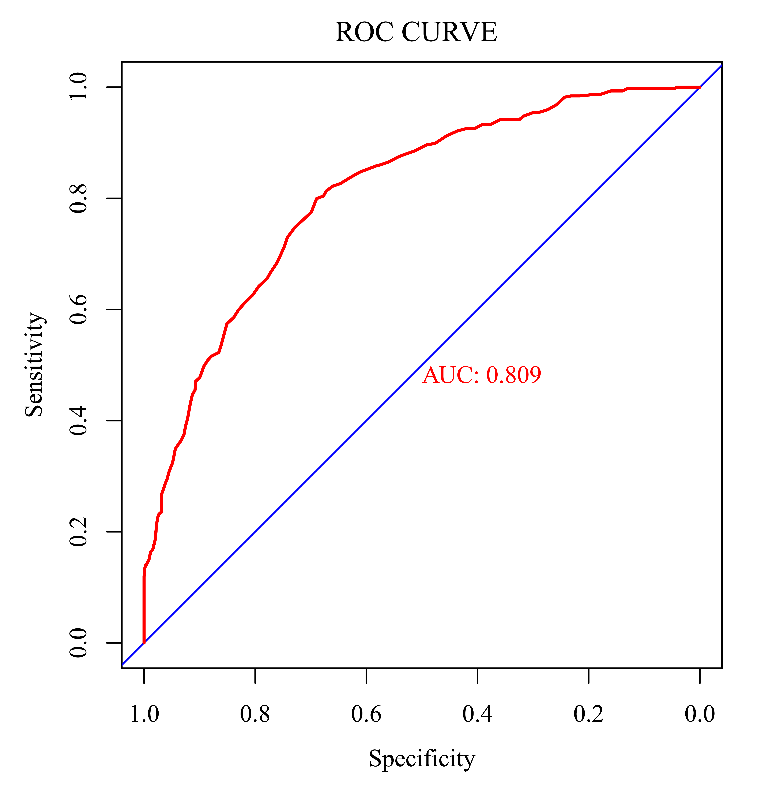


Figure S3. ROC curve of PSI in SCAP patients with T2DM. ROC: receiver operating characteristic; PSI: Pneumonia Severity Index.
